# Epigenetic Alterations in Microbiome–Host Interactions in Inflammatory and Autoimmune Diseases

**DOI:** 10.3390/ijms27083354

**Published:** 2026-04-08

**Authors:** Abdallah A. Basher, Mokhtar Rejili, Abdelkareem A. Ahmed, Mohamed Osman Abdalrahem Essa, Nasir A. Ibrahim, Nosiba S. Basher, Hosameldeen Mohamed Husien, Ahmed A. Saleh, Mudathir Y. Abdulrahman, Rifat Ullah Jan, Saber Y. Adam, Demin Cai

**Affiliations:** 1 Jiangsu Key Laboratory of Animal Genetic Breeding and Molecular Design, College of Animal Science and Technology, Yangzhou University, Yangzhou 225009, China; mh24012@stu.yzu.edu.cn (A.A.B.); 008643@yzu.edu.cn (H.M.H.); elemlak1339@yzu.edu.cn (A.A.S.); mudathir@duc.edu.sd (M.Y.A.); dh23053@stu.yzu.edu.cn (R.U.J.); saber@duc.edu.sd (S.Y.A.); 2 Department of Biology, College of Science, Imam Mohammad Ibn Saud Islamic University (IMSIU), Riyadh 11623, Saudi Arabia; msrejili@imamu.edu.sa (M.R.); nsbasher@imamu.edu.sa (N.S.B.); 3 Biomedical Research Institute, Darfur University College, Nyala 155, Sudan; aabdallah@buan.ac.bw; 4 Department of Veterinary Sciences, Faculty of Animal and Veterinary Sciences, Botswana University of Agriculture and Natural Resources, Gaborone 0027, Botswana; 5 College of Veterinary Medicine, Yangzhou University, Yangzhou 225009, China; dh23054@stu.yzu.edu.cn; 6 College of Veterinary Medicine, Albutana University, Rufaa 22217, Sudan; 7 Animal and Fish Production Department, Faculty of Agriculture (Al-Shatby), Alexandria University, Alexandria 11865, Egypt

**Keywords:** epigenetic, host microbiome, inflammatory disorders, autoimmune diseases

## Abstract

Inflammatory and autoimmune diseases are now understood to be significantly influenced by the intricate interactions between the microbiome and host physiology. This review investigates the function of epigenetic dysregulation in microbiome–host interaction and its consequences for health and disease. Epigenetic modifications, including DNA methylation, histone modifications, and non-coding RNA-associated regulation, are key mechanisms that control gene expression without altering the underlying DNA sequence. Microbial metabolites and community composition alterations can cause disruptions in these epigenetic processes, resulting in dysregulated immune responses and the initiation of chronic inflammatory conditions. In particular, the diversity of gut microbiota alters host epigenetic patterns, affecting T cell differentiation, inflammatory modulation, and tissue homeostasis. Aberrant epigenetic modifications contribute to the pathogenesis of autoimmune diseases such as rheumatoid arthritis (RA) and systemic lupus erythematosus (SLE) by promoting inflammation and autoimmunity. Similarly, gut microbiota dysbiosis has been implicated in the development and progression of inflammatory bowel disease (IBD). Identifying the reciprocal interaction between epigenetic alterations and microbiome dynamics provides unique insights into therapeutic options targeted at restoring microbial homeostasis to prevent disease progress. Consequently, understanding the intricacies of epigenetic dysregulation in microbiome–host interactions represents a significant sector in biomedical research and highlights the promise for precision medicine approaches in treating inflammatory and autoimmune diseases. The potential for microbiome-based therapies to affect host epigenetic landscapes requires additional research, paving the way for innovative therapeutic paradigms targeted at improving host resilience and restoring immunological balance. The purpose of this review is to synthesize current knowledge on how epigenetic dysregulation and microbiome–host interactions drive inflammatory and autoimmune diseases and to highlight emerging therapeutic opportunities.

## 1. Introduction

The gastrointestinal tract (GIT) is a complex ecosystem populated by trillions of commensal fungi, archaea, viruses, and bacteria, collectively termed the microbiota [1]. The GIT contains around 70% of the human microbiota, and its composition varies depending on the anatomical location, with density increasing from the proximal to the distal gut [2]. *Fusobacteria*, *Actinobacteria*, *Proteobacteria*, and *Verrucomicrobia* are the most common microbial phyla in the gut, followed by *Firmicutes* and *Bacteroidetes* [3], making up 90% of gut microbiota. The gut microbiota is vital for maintaining host health because it improves the host’s digestive ability, protects the intestinal epithelial barrier, prevents pathogen entry, improving immunity, and tissue development [4] by producing some important neurotransmitters [5], vitamins, and hormones [6]. Additionally, byproducts of the gut microbiota have the ability to modify host physiology and metabolism, thereby facilitating food digestion and energy production from indigestible substrates [7]. For example, short-chain fatty acids (SCFAs) are produced from indigestible fiber. SCFAs fuel the intestinal mucosa, maintaining homeostasis and regulating immune responses to prevent inflammation and carcinogenesis [8,9]. Importantly, however, alterations in the amount of microbiota or composition have been associated with chronic health problems in humans, including cancer, metabolic diseases, and inflammatory bowel disease (IBD) [4,10].

The term “epigenetic” indicates alterations to the genome that could be heritable and reversible, without changing DNA sequences [11]. In multicellular animals, these modifications allow the single genome to generate different cell/tissue types or change its transcriptional repertoire to the constantly changing conditions of the environment [12]. These processes regulate gene expression through chromatin structure modulation or regulating transcriptional apparatus binding to DNA. An important feature of these mechanisms is their susceptibility to being modulated by a wide range of factors, including pathological and physiological stimuli, in addition to environmental factors such as work habits, physical activity, stress, diet, alcohol consumption and smoking [13,14]. Epigenetic dysregulation is one of the most common epigenetic distresses in diseases. This dysregulation plays a vital role in disease development by manipulating cell growth, differentiation, metabolism, apoptosis, and signaling [15]. Protein structure and gene expression may change as a result of abnormal DNA methylation and post-translational modifications (PTMs), which eventually accelerate the development of disease. Epigenetic markers like mA, 5-mC, and 5-hmC are essential in biological processes and their abnormal regulation is associated with various diseases [16], including inflammatory and autoimmune diseases.

Inflammatory disorders encompass a broad range of conditions characterized by dysregulated inflammation, affecting both acute and chronic disease states. Inflammation arises from the immune system’s response to harmful stimuli and can contribute to the pathogenesis of several autoimmune diseases, including rheumatoid arthritis (RA) [17], inflammatory bowel disease (IBD), and systemic lupus erythematosus (SLE) [18].

The progression of autoimmune diseases is often conceptualized in three overlapping stages. First, a loss of immunological tolerance occurs, triggered by genetic susceptibility and environmental factors (e.g., infections and microbiome alterations). During this stage, self-reactive T and B cells escape normal regulatory mechanisms, leading to aberrant immune activation. Second, the breakdown of tolerance gives rise to persistent inflammation and tissue damage. Innate immune cells (macrophages, granulocytes, and dendritic cells) proliferate and secrete pro-inflammatory mediators, which in turn recruit and activate adaptive immune cells, amplifying the autoimmune response. Third, a regulatory phase may ensue, during which immune regulation attempts to restrain inflammation; however, in established autoimmune diseases, this phase is often incomplete, resulting in chronic relapsing inflammation [19,20].

The objective of this review is to provide a conceptual framework for understanding how epigenetic dysregulation and microbial communication induce autoimmune and inflammatory diseases. Furthermore, we discuss prospective strategies for the implementation of microbial therapies in therapeutic approaches to autoimmune and inflammatory diseases, and potential mechanisms by which epigenetic dysregulation and the host microbiota influence the pathogenesis of these diseases.

## 2. Epigenetic Mechanisms in the Microbiome–Host Interaction

### 2.1. DNA Methylation

DNA methylation represents a major epigenetic regulatory mechanism in humans, influencing gene expression by modulating transcription factor accessibility to DNA. Epigenetic machinery includes semi-conservative maintenance of methylation patterns during DNA replication, primarily facilitated by proteins such as DNMT1 that recognize hemimethylated CpG sites and methylate the newly synthesized strand [21].

DNA methyltransferases (DNMTs) catalyze the addition of a methyl group from S-adenosylmethionine (SAM) to cytosine, forming 5-methylcytosine (5mC). DNMT1 maintains existing methylation patterns during cell division, whereas DNMT3A and DNMT3B establish de novo methylation. Active DNA demethylation is mediated by ten-eleven translocation (TET) enzymes, which oxidize 5mC to 5 hydroxymethylcytosine (5hmC) in an α ketoglutarate-dependent manner [22]. Dysregulation of DNA methylation is a hallmark of many inflammatory and autoimmune diseases, and the gut microbiota influences these patterns through production of methyl donor precursors (folate and methionine) and S adenosylmethionine (SAM), linking microbial metabolism directly to the host epigenome [23,24,25].

**Figure 1:**
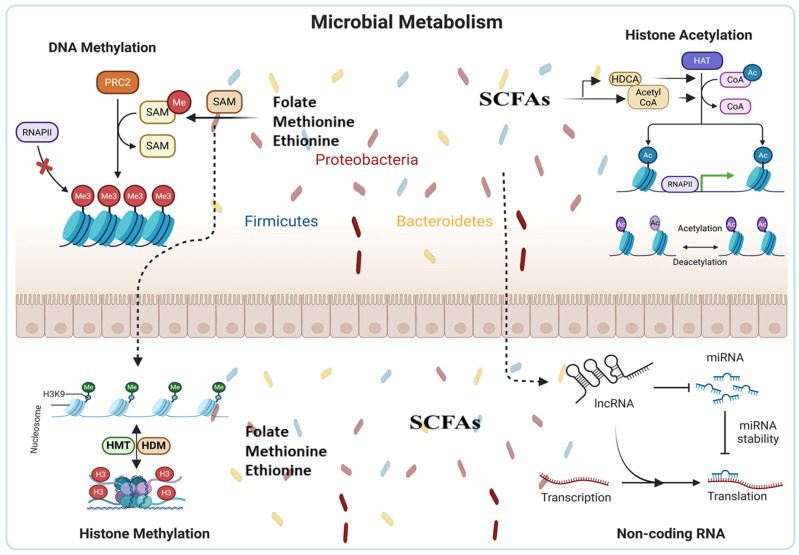
Schematic overview of microbiota–host epigenetic crosstalk. Gut microbiota and their metabolites (for example, short-chain fatty acids (SCFAs), folate, methionine, and secondary bile acids) influence host epigenetic machinery through multiple routes. Left panel: Microbial metabolites serve as donors or cofactors for DNA methyltransferases (DNMTs) and histone methyltransferases (HMTs) via the one-carbon metabolism pathway (folate, S-adenosylmethionine (SAM)), or act as histone deacetylase (HDAC) inhibitors (e.g., butyrate), thereby modulating DNA methylation and histone acetylation patterns. Center panel: Metabolites can also be incorporated as acyl-CoA precursors, providing acetyl groups for histone acetyltransferases (HATs). Right panel: Gut microbiota influence the expression of non-coding RNAs (ncRNAs), including microRNAs (miRNAs) and long non-coding RNAs (lncRNAs), which in turn regulate gene expression post-transcriptionally. The resulting epigenetic changes affect key host processes such as immune cell differentiation, inflammatory signaling, and intestinal barrier integrity, thereby contributing to either health or disease susceptibility. Created in BioRender. Ahmed, N. (2026) https://BioRender.com/v968e5h (accessed on 6 January 2026).

Gut bacteria can alter host DNA methylation patterns by producing epigenetically active metabolites, including SCFAs. These metabolites support DNA methylation processes. In particular, folate is essential for one-carbon metabolism and the synthesis of SAM, the primary methyl donor for DNMTs. A coculture of human fetal and adult intestinal epithelial cells with *Bifidobacterium infantis* and *Lactobacillus acidophilus* induces significant alterations in DNA methylation patterns and elicits a bacteria-specific transcriptomic response, underscoring this mechanism [23,24]. A summary of key microbial metabolites and their established epigenetic targets is provided in Table 1. In germ-free (GF) mice and conventionally housed controls, global DNA methylation levels in the intestinal epithelium are significantly reduced in GF hosts compared to conventionally colonized mice [25]. This hypomethylation is primarily attributed to diminished availability of microbiota-derived one-carbon metabolites, rather than reduced DNMT activity. As illustrated in Figure 1, microbial metabolites such as folate, methionine, and SCFAs serve as donors or regulators for DNA methylation and histone modifications, thereby linking microbial metabolism directly to host epigenetic machinery.

### 2.2. Histone Modifications

Histone modifications represent a key epigenetic mechanism that regulates critical chromatin-associated processes, including transcription, DNA repair, and replication. Histones are classified into five main families: the linker histones H1 and H5, and the core histones H2A, H2B, H3, and H4 [26,27]. Linker histones (H1/H5) bind to DNA between nucleosomes to facilitate higher-order chromatin folding, whereas core histones (two each of H2A, H2B, H3, and H4) form the octameric nucleosome core around which DNA is wrapped. These histones undergo diverse PTMs, such as acetylation, methylation, phosphorylation, ubiquitination, and SUMOylation, which dynamically alter chromatin structure and function [28]. Figure 1 shows the interaction of microbial metabolism and histone modifications.

#### 2.2.1. Histone Acetylation

Histone acetylation, governed by histone acetyltransferases (HATs) and histone deacetylases (HDACs), is generally associated with transcriptional activation. HATs transfer acetyl groups from acetyl-CoA to lysine residues, neutralizing their positive charge and relaxing chromatin structure, while HDACs remove acetyl groups, promoting condensation and transcriptional repression [29,30,31,32]. Active promoters and enhancers are enriched in histone H3 acetylation at lysine 9 (H3K9ac) and lysine 27 (H3K27ac), which facilitates RNA polymerase II transition from pausing to elongation [33,34]. Acetylation also occurs in the histone H3 core (e.g., H3K56ac), affecting histone–DNA interactions [35].

It has long been recognized that histone acetylation in the intestine is influenced by dietary fiber intake and the production of SCFAs by the gut microbiota. As summarized in Table 1, SCFAs such as butyrate act as HDAC inhibitors and also serve as acetyl-CoA precursors, thereby modulating histone acetylation patterns in host cells. To dissect the microbiota’s role, the investigators utilized germ-free (GF), conventionally reared, and microbiota-recolonized (“conventionalized”) mice. The presence of gut microbiota across multiple organs markedly increased acetylation of histones H3 and H4 at various lysine residues, whereas changes in H3 methylation were more subtle yet statistically significant [36]. Mechanistically, the gut microbiota modulates the expression of genes encoding enzymes involved in the generation of metabolites essential for histone PTMs. For instance, expression of ATP citrate lyase (Acly), an enzyme critical for glucose-derived (but not acetate-derived) histone acetylation in mammalian cells, was significantly lower in conventionally raised mice compared to GF controls under both chow and high-fat/high-sugar (HF/HS) diets. This indicated that the suppression of glucose-driven histone modification may occur when bacterial SCFA is present. SCFAs such as butyrate can increase histone acetylation by inhibiting HDACs [37,38]. This mechanism affects immune cell differentiation and metabolic regulation, among other biological processes. Furthermore, SCFAs can act as precursors of acyl-CoA, facilitating histone acetylation by providing acetyl groups for HATs. Histone acetylation modulation by the microbiota, illustrated in Figure 1, emphasizes the complex interplay between host epigenetics and gut microorganisms that influences health and disease susceptibility.

Beyond HATs and HDACs, chromatin ‘readers’ that recognize acetylated histones play a critical role in translating acetylation signals into gene expression outcomes. Bromodomain and extra-terminal domain (BET) proteins (such as BRD2, BRD3 and BRD4) bind acetylated lysines on histones and recruit transcriptional regulators, thereby promoting the expression of pro-inflammatory genes. BET inhibitors have shown therapeutic potential in preclinical models of inflammatory diseases, including colitis and rheumatoid arthritis, by suppressing key inflammatory mediators [39,40].

**Table 1:** Selected microbial metabolites and their epigenetic targets.

Metabolite	Source/Producing Bacteria	Epigenetic Target/Mechanism	Effect on Host Cells/Outcome	Ref.
Butyrate	*Clostridia*, *Faecalibacterium prausnitzii*, *Roseburia* spp.	HDAC inhibition; histone acetylation (H3K9ac, H3K27ac); promoter acetylation	Treg expansion; anti-inflammatory gene expression; intestinal barrier integrity	[36,37,41,42]
Acetate, Propionate	Various *Bacteroidetes*, *Bifidobacterium*	HDAC inhibition; GPR41/GPR43 signaling; histone acetylation	Modulation of immune cell function; Treg differentiation; anti-inflammatory effects	[36,43,44]
Folate	*Bifidobacterium*, *Lactobacillus*	Substrate for one-carbon metabolism; SAM synthesis; DNA methylation	Maintenance of DNA methylation patterns; intestinal epithelial cell function	[23,24]
Methionine/S-adenosylmethionine (SAM)	Gut microbiota (via dietary methionine)	Methyl donor for DNMTs and histone methyltransferases	DNA methylation; histone methylation (H3K4me, H3K9me, H3K27me)	[23,45]
Ethionine (analog)	Microbial metabolism	Interference with SAM-dependent methylation	Altered DNA and histone methylation; potential pro-inflammatory effects	[22]
Secondary bile acids	*Clostridium* spp., *Eubacterium*	Regulation of histone acetylation via HDAC inhibition; modulation of nuclear receptors	Regulation of metabolic and inflammatory gene expression	[22,46]

#### 2.2.2. Histone Methylation

Histone methylation, catalyzed by lysine methyltransferases (KMTs) using S-adenosylmethionine (SAM) as a methyl donor, can either activate or repress transcription depending on the residue and methylation state. For example, H3K4me3 is associated with active gene promoters, whereas H3K9me3 and H3K27me3 mark transcriptionally silent regions. Dynamic regulation by histone demethylases (KDMs) adds another layer of complexity. Notably, the availability of SAM and the ratio of SAM to S-adenosylhomocysteine (SAH) can be influenced by microbial metabolism, linking diet, gut microbiota, and histone methylation [45,47].

#### 2.2.3. Histone Phosphorylation

There are two types of enzymes that regulate the status of histone phosphorylation, although their methods of action are different [48]. Using ATP as a phosphate group donor, kinases such as KAT2A phosphorylate the hydroxyl group of the amino acids serine, tyrosine, and threonine [35,49]. Phosphorylated histones have at least three recognized functions: they repair damage to DNA, regulate transcriptional activity (much like histone acetylation), while controlling the chromatin compaction linked to meiosis and mitosis [35,50]. Unlike histone acetylation and methylation, histone phosphorylation works in tandem with other histone modifications, establishing the foundation for their reciprocal interactions [50]. This crosstalk produces complex downstream chromatic status modulation and its effects [35]. Histone H3 phosphorylation, in particular, H3S10ph, has the ability to directly influence the amounts of acetylation at two positions of amino acids within the same histone, specifically H3K9ac and H3K14ac [51]. Moreover, the interaction of H3S10ph with H4K16ac can induce transcriptional activation [52]. H2AXS139ph induces DNA damage repair proteins to the location of double-stranded DNA breaks, which is another instance of histone phosphorylation regulation [53,54].

### 2.3. Non-Coding RNAs (ncRNAs)

Beyond DNA and histone modifications, non-coding RNAs (ncRNAs) constitute another layer of epigenetic regulation. Long non-coding RNAs (lncRNAs) and microRNAs (miRNAs) do not encode proteins but modulate gene expression post transcriptionally by influencing mRNA stability, translation, or chromatin architecture [55]. Importantly, ncRNAs serve as critical mediators in microbiome–host crosstalk: microbial metabolites can alter miRNA expression, and host-derived miRNAs can in turn shape gut microbial composition [56,57,58,59,60,61]. This bidirectional interaction adds a dynamic dimension to the epigenetic landscape, linking environmental microbial signals to host gene regulation.

lncRNAs in particular have been shown to be useful in differentiating between different types of gut microbiota and in serving as biomarkers to identify the host–microbiota interaction process [56]. The expression of lncRNAs in mice with and without microflora is discovered to be distinctive. Mice colonized with a particular strain of bacteria showed a significantly different lncRNA profile when compared to GF mice; the majority of these lncRNAs were transcribed from introns [57,58]. Furthermore, colonization with wild-type *E. coli* or *E. coli*-producing bile salt hydrolase had differing impacts on lncRNA expression [22]. The changes observed in host epithelial cells were bacteria-species-dependent, despite the fact that a small fraction of the detected lncRNAs correlated with known lncRNAs related to inflammation. The expression of lncRNAs can be regulated by the gut microbiota, which can impact downstream target molecules and ultimately host transcriptomes [58,59]. Furthermore, lncRNAs have an important role in the microbiome–host crosstalk by interacting with microbial components to maintain intestinal homeostasis and protect against colitis [60]. miRNAs can also regulate commensal microbiota-dependent intestinal epithelial cells, which maintain gut dysbiosis and homeostasis. miRNAs from host fecal samples can infiltrate bacteria, such as *F. nucleatum* and *E. coli*, and hence influence bacterial gene transcription and growth. In support of this finding, the coculture of miRNAs with bacteria can induce considerable changes in bacterial gene expression [61]. These data reveal that the host releases miRNA feedback onto the gut bacteria to maintain the intestine’s homeostasis [22]. Understanding the processes via which non-coding RNAs work in the microbiome–host relationship offers valuable insights into prospective therapeutic approaches for numerous infections and disorders. Figure 1 shows the interaction of microbial metabolism and Non-coding RNAs.

While the roles of DNA methylation, histone modifications, and ncRNAs in microbiome–host interactions are well established, several knowledge gaps remain. First, the causal relationships between specific microbial taxa and particular epigenetic marks are often inferred from correlative studies; experimental validation using gnotobiotic models and epigenetic editing is still limited. Second, the effects of microbial metabolites on epigenetic enzymes can be context dependent. For example, butyrate acts as an HDAC inhibitor in most cell types but may have opposite effects under certain metabolic conditions [38]. Third, the cell type specificity of epigenetic responses to microbial signals remains poorly characterized, highlighting the need for single cell epigenomic approaches. Addressing these gaps will be crucial for translating mechanistic insights into therapeutic strategies.

## 3. Impact of Epigenetic Dysregulation on the Microbiome–Host Interaction

### 3.1. Dysregulated Immune Response in Host

The gut microbiota is essential for the normal development and function of both local and systemic immune responses. Recent evidence has linked immune cell regulation to epigenetic changes driven by microbial metabolites and community composition. This section synthesizes findings across key immune cell types, such as regulatory T (Treg) cells, innate lymphoid cells (ILCs), natural killer (NK) cells, and intestinal epithelial cells (IECs), to illustrate how microbiota-induced epigenetic alterations shape immune function and contribute to disease.

#### 3.1.1. Regulatory T (Treg) Cells

Tregs maintain immune tolerance by suppressing aberrant CD4^+^ T cell activation against commensal bacteria, and their numbers are significantly reduced in the colonic lamina propria of GF mice [62]. Short-chain fatty acids (SCFAs) produced by gut microbiota, particularly butyrate from *Clostridia* strains, promote Treg expansion and function through epigenetic mechanisms [43,63,64]. Butyrate inhibits HDAC activity [65,66], leading to increased H3K27 acetylation at the *Foxp3* promoter and its CNS-1 enhancer, thereby facilitating Treg differentiation [41]. Simultaneously, butyrate suppresses pro-inflammatory genes such as *IL-6*, *IL-12*, and *Relb* [41]. These epigenetic effects are cell-intrinsic and depend on microbial colonization, as demonstrated by increased colonic Treg proliferation in colonized versus GF mice [67]. SCFAs also induce histone hyperacetylation at the *p21Waf1/Cip1* promoter, affecting cell cycle regulation [42]. Collectively, SCFA-mediated epigenetic regulation of Tregs exemplifies how microbiota-derived metabolites directly influence immune tolerance.

#### 3.1.2. Innate Lymphoid Cells (ILCs)

ILCs orchestrate inflammation and tissue homeostasis at mucosal barriers. Their development and function are governed by epigenetic programs [68] and are highly responsive to gut microbiota. In terms of effector cytokine profiles and transcription factor requirements, ILCs and T cells share similarities in coordinating inflammatory responses at mucosal sites [69]. Gut microbiome colonization influences ILC composition, development, and function, either directly or indirectly [70,71]. Transcriptomic and epigenomic analyses in GF or antibiotic-treated mice revealed that ILC1s and ILC2s are more affected by microbiota depletion than ILC3s, with thousands of H3K4me2 sites altered and a loss of subset-specific transcriptional identity [72]. These changes result from microbiota-induced reprogramming of enhancer landscapes, which in turn influences downstream transcription factors critical for intestinal immunity and colon cancer development. Thus, the microbiota shapes the epigenetic landscape of ILCs in a subset-specific manner, linking microbial signals to innate immune function.

#### 3.1.3. Natural Killer (NK) Cells

NK cells, derived from NK/T cell progenitors and maturing in bone marrow and secondary lymphoid tissues [73], play key roles in antiviral and antitumor immunity and have been implicated in autoimmune diseases such as systemic lupus erythematosus [74]. Their effector function depends on gut microbiota: in GF or antibiotic-treated mice, NK cells fail to mount effective cytotoxic responses against cytomegalovirus (MCMV) due to impaired priming by dendritic cells (DCs) [75,76]. Mechanistically, virus-induced activation of DCs in colonized mice leads to rapid recruitment of NF-κB-p65, IRF3, and RNA polymerase II to the promoters of *Ifnb1*, *Tnf*, and *IL-6*, whereas GF mice lack this transcriptional activation. This defect is likely due to a chromatin-level deficiency arising from the absence of microbiota-induced epigenetic priming. Notably, reconstitution of the microbiota restores NK cell functionality, underscoring the essential role of microbial signals in establishing an epigenetically permissive state for NK cell effector genes.

#### 3.1.4. Intestinal Epithelial Cells (IECs)

IECs form a physical and functional barrier between the gut lumen and the immune system, with key roles in segregation and mediation [77,78]. Their epigenetic state is dynamically regulated by the microbiota. For example, commensal bacteria induce DNA hypomethylation in the *TLR4* gene promoter, modulating inflammatory responses [79,80]. IECs from IBD patients show reduced HDAC3 expression, and HDAC3 deletion in IECs from GF mice does not affect barrier function, indicating that microbial signals are required for HDAC3-mediated transcriptional regulation [81]. Pathogens can also alter the IEC epigenome; *H. pylori* infection induces hypermethylation of the DNA repair gene *MGMT*, promoting gastric inflammation and mutagenesis [82,83,84]. Together, these examples illustrate that both commensal and pathogenic bacteria shape IEC epigenetic landscapes, with direct consequences for barrier integrity and inflammation.

### 3.2. Altered Gene Expression in Microbiota

Antibiotic treatment of high-fat diet (HFD)-fed obese mice induces marked shifts in gut microbiota composition, characterized by an increase in *Proteobacteria* and a concomitant decrease in *Firmicutes* and *Actinobacteria*. These microbiota alterations were associated with changes in promoter DNA methylation patterns that upregulated mRNA expression of genes involved in lipid oxidation, as well as resistin and adiponectin pathways [85]. In parallel, a genome-wide methylation analysis of DNA from stool, blood, and adipocytes in 45 obese individuals identified significant differential methylation at 258 genes between groups stratified by low versus high *Bacteroides-to-Firmicutes* ratio. Moreover, gut microbiota may exert epigenetic regulation on candidate genes such as HDAC7 and IGF2BP2, which are implicated in glucose homeostasis and energy metabolism [86].

Interactions between the gut microbiota and dietary components can remodel histone modifications at active enhancers, thereby influencing downstream transcription factor activity [87]. In vivo studies in mice have shown that histone methylation—particularly activating marks such as H3K4me or repressive marks like H3K9me and H3K27me mediates transcriptional dysregulation that contributes to obesity and hyperlipidemia [88]. *Prevotella* abundance is strongly associated with altered methylation of AFAP1, offering a potential epigenetic mechanism to enhance insulin sensitivity and ameliorate metabolic syndrome [89]. Consumption of resistant starch type 4 (RS4) in humans increases fecal SCFA levels particularly butyrate and enriches butyrate-producing bacteria [90]. In RS4-fed mice, colon tissue exhibits elevated trimethylation of H3K27me3 at the promoter of NFκB1, suggesting microbiota-driven repressive epigenetic control of inflammatory pathways [91]. HDAC3, highly responsive to microbial signals, regulates histone acetylation in IECs and is essential for intestinal homeostasis [81,92]. Beyond the gut, HDAC3 also governs lipid homeostasis in muscle, liver, and adipose tissue. Paradoxically, while butyrate inhibits HDAC3 activity in IECs to protect against diet-induced obesity, epithelial HDAC3 promotes the development of diet-induced obesity in vivo [93]. In HDAC6-deficient mice, gut microbiota composition shifts toward reduced S24-7 family members and *Lactobacillus* spp., with corresponding increases in *Bacteroides* and *Parabacteroides*. These changes implicate HDAC6 in modulating microbiota-dependent enhancement of Treg suppressive function and attenuation of inflammatory responses [94].

Small endogenous ncRNAs called miRNAs supplement mRNA bases, control post-transcriptional gene expression, encourage mRNA decay, or inhibit translation, among other biological activities [95]. IECs secrete the majority of miRNAs in the ileum, which are subsequently found in feces [61,96]. miRNAs interact with microbiota to modulate the host’s gene expression through post-transcriptional regulation [97,98]. The place or interacting region where miRNAs and their target mRNA interact determines whether post-transcriptional gene expression is up or downregulated [99,100]. Similarly, the connection between intestinal microbiota and the miRNAs present in the intestinal contents is accomplished based on controlling gene transcripts of microbial cells through miRNA-targeted complementary nucleic acid sites [101]. Research by Liu et al. [102] has shown that host cells can influence the composition of the intestinal microbiota via the miRNA pathway. Specifically, fecal miRNAs can directly regulate the expression of certain bacterial genes and affect microbial growth in the gut. In mice lacking miRNAs in IEC, the gut microbiota undergoes significant changes. Notably, the miR-515-5p and miR-1226-5p are capable of entering bacterial cells, co-localizing with bacterial nucleic acids, and promoting the growth of specific bacteria linked to colorectal cancer (CRC), such as *Fusobacterium nucleatum* and *Escherichia coli*, by altering their gene expression [102]. Conversely, some host-derived miRNAs have been found to suppress microbial proliferation in animal studies. For example, human miR-21 directly inhibits the growth of *Lactobacillus reuteri* in vitro, and mice lacking CRC-associated miR-21 exhibit markedly increased levels of Lactobacillus species in their intestines [103]. The role of the fungal microbiome in CRC development has received less attention but is also relevant. Studies have shown that the pathogenic fungus *Candida albicans* interacts with human monocytes, triggering the production of a host miRNA that enhances fungal growth. This finding highlights an unexpected cross-kingdom interaction and suggests that targeting or reducing certain miRNAs could offer new therapeutic avenues for fungal infections [104]. Further research is needed to clarify the mechanisms by which miRNAs enter bacterial cells and the subsequent processing that occurs inside them, as different miRNAs vary in their ability to penetrate bacteria and thus differ in their regulatory impact.

### 3.3. Adjustment of the Intestinal Homeostasis

Through regulating the gut microbiota, host miRNAs can contribute to physiological processes associated with preserving intestinal homeostasis [31]. For instance, for IECs, miR-21-5p expression can regulate intestinal epithelial barrier permeability by influencing ADP ribosylation factor 4 (ARF4) [105]. A comprehensive intestinal barrier against enteropathogens is established by the gut microbiota, intestinal epithelium, and mucosal system [106]. Together, these various populations of intestinal epithelial cells create a barrier that keeps gut bacteria contained within the intestinal lumen. In addition, epithelial cells act as a conduit for information between immunocompetent cells situated in the lamina propria, the GALT’s effector site. They do this by transmitting signals between cells directly or through mediators (cytokines and chemokines), which primes the immune system or promotes immunological tolerance. Multiple epigenetic processes affect the development of IECs the quantity of gut bacteria, and the metabolites produced by them [46]. According to related research, host-secreted miRNAs regulate the formation and composition of microbial communities, which may have a significant effect on intestinal homeostasis and provide a novel approach to preserving intestinal health [107]. Additional study on this area is needed in the future because it is currently unclear how these epigenetic elements interact with diverse cues from the symbiotic bacteria.

### 3.4. Dysregulation of the Host Metabolism

The term “metabolism” refers to a broad range of distinct, organized chemical processes that organisms undergo in order to meet their basic needs for energy production and protein synthesis, among other things. These mechanisms help organisms stay alive and respond to their environment [108]. miRNAs play an important part in the intricate communication between the gut bacteria and the host. Current evidence indicates that circulating miRNA and gut bacteria help to provide a significant pathway for obesity. In the obese group, 26 distinct circulating miRNAs and 12 microbial species exhibited differential abundance compared to the control group, with significant associations observed between these miRNAs and microorganisms. Specifically, three miRNAs implicated in the modulation of body mass index (BMI) namely (miR-130b-3p, miR-185-5p, and miR-21-5p) demonstrated a negative correlation with the abundance of *Bacteroides eggerthii*. Additionally, the expression levels of miR-107, miR-103a-3p, miR-222-3p, and miR-142-5p were inversely associated with the abundance of *Bacteroides intestinihominis*.

These miRNAs are known to regulate target genes involved in key metabolic pathways, including insulin signaling, fatty acid β-oxidation, and glycerolipid metabolism. In particular, miR-15a promotes insulin production by suppressing the expression of uncoupling protein 2 (UCP2), which in turn elevates cellular ATP levels and enhances glucose-stimulated insulin secretion. In vivo studies have revealed an inverse relationship between the abundance of *Haemophilus parainfluenzae* and the expression level of miR-15a-5p, suggesting that this bacterial species may influence circulating insulin concentrations through its association with miR-15a-5p.

Furthermore, a bioinformatic analysis identified significant differences in obese individuals compared to controls, both in the abundance of four specific gut bacterial taxa (*Dorea longicatena*, *Bacteroides intestinihominis*, *Bacteroides eggerthii*, and *Haemophilus parainfluenzae*). Correlation analyses further supported the existence of direct or indirect interactions between these microbial taxa and the dysregulated miRNAs, pointing to a bidirectional crosstalk mechanism between the gut microbiota and host miRNA profiles in obesity [109]. Resident bacteria are impacted by miRNAs secreted in the intestinal lumen, demonstrating the intimate connection between miRNAs and gut microbiota that is necessary for intestinal homeostasis and maturation [110]. The gut microbiota affects miRNAs, which help to modulate intestinal immune responses and may have an impact on the development of intestinal immunological disorders that include IBD. Nevertheless, additional research is required to fully understand the mechanism, which is vital for developing individualized diets and precision nutrition therapies.

## 4. Impact of Epigenetic Dysregulation in Inflammatory Disorders and Autoimmune Diseases

The convergence of microbiome dysbiosis, epigenetic alterations, and aberrant immune responses lies at the heart of many chronic inflammatory and autoimmune diseases. In this section, we discuss three prototypical conditions IBD, RA and SLE in which the interplay between microbial factors, epigenetic modifications, and immune dysregulation has been most extensively studied. A comparative overview of the major epigenetic alterations observed in IBD, RA, and SLE is presented in Table 2, highlighting shared pathways and disease specific features.

### 4.1. Inflammatory Bowel Disease (IBD)

**Figure 2:**
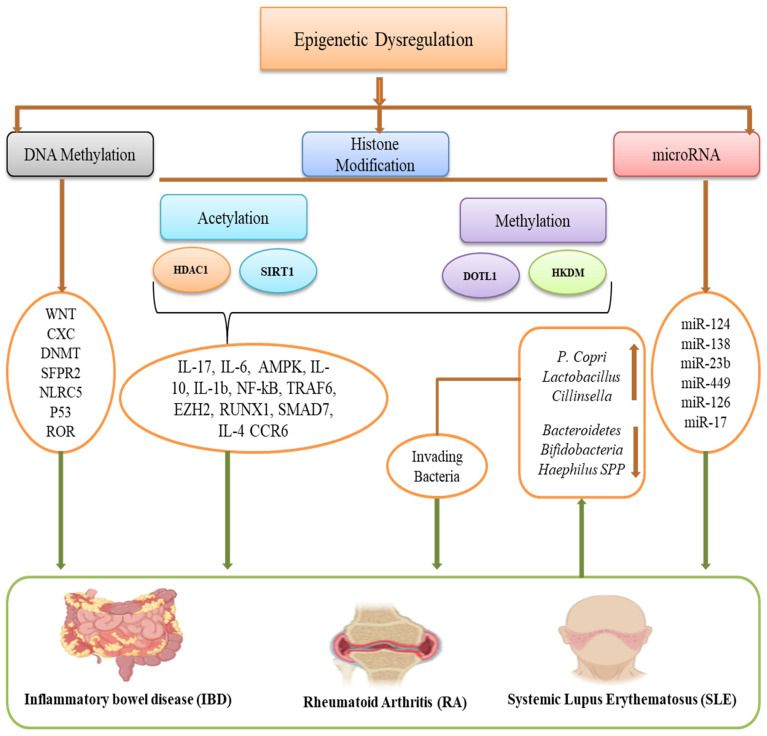
A schematic diagram of the epigenetic dysregulation (DNA methylation, histone modification, and miceiRNA) mediating IBD, RA, and SLE progression. Acetylation (HDAC1, SIRT1), and methylation (DOTL1, HKDM) of disease-related proteins mediates the expression of disease factors (IL-17, IL-6, AMPK, IL-10, IL-1b, NF-kB, TRAF6, EZH2, RUNX1, SMAD7 IL-4, CCR6, etc.), thereby affecting the proliferation, invasion, and apoptosis of disease-related cells. (Created in BioRender. Ahmed, N. (2026) https://BioRender.com/ubeglqk) (accessed on 6 January 2026).

IBD is a chronic, recurrent inflammatory bowel disease linked to an increased risk of colon cancer. It also comprises Crohn’s disease (CD) and ulcerative colitis (UC) [111,112]. Every year, the incidence of UC is larger than that of CD. The most common diagnosis for UC happens in the third and fourth decades of life, while CD is mainly detected in the second and third decades [113]. The country’s economic development and geographic location have an impact on the incidence of IBD. They can be found in North America, China, South Korea, Japan, India, and Australia, but they are primarily found in Europe, especially in Scandinavia and the UK [114]. IBD’s causes are not entirely understood, though numerous investigations indicate that immunological, genetic, microbiological, and environmental variables are present in IBD and interact with one another [115]. Epigenetics most certainly contribute to the pathophysiology of IBD, and the gut microbiota plays a pivotal role in shaping these epigenetic landscapes. As summarized in Table 2, the interplay between microbial dysbiosis, epigenetic alterations, and immune dysregulation is central to disease pathogenesis [116]. Figure 2 shows the role of epigenetic dysregulation in IBD.

#### 4.1.1. DNA Methylation in IBD

The most prevalent and most researched epigenetic direction of IBD is DNA methylation. Research has demonstrated that individuals suffering from CD and UC display worldwide hypomethylation of DNA in multiple cell types, such as immune cells and IECs [117,118,119,120]. Microbiota composition changes, particularly reductions in butyrate-producing Clostridia and Faecalibacterium prausnitzii, are linked to the altered availability of methyl donors (folate and SAM) and to specific methylation changes, such as hypomethylation of RPS6KA2 [118] and hypermethylation of anti-inflammatory genes like *IL-10* [120]. Probiotic *Bifidobacterium longum* and melatonin may affect the methylation status of DNA in IECs [121,122]. Pan et al. [123] provided evidence of the microbiota’s impact on transcriptome alterations and the maturation of DNA methylation characteristics. They revealed that it controls the transcriptome of the gut during postnatal development and targets a subset of genes that react to the microbiota through the matching DNA methylation status [123]. According to Ansari et al., the microbiota modulates the activation of particular genes linked with hypomethylated active regulatory regions, which promotes the expression of genes involved in IBD and colitis [124].

#### 4.1.2. Histone Modification in IBD

Histone modifications are critical for the development and progression of IBD. For example, phosphorylation of histone H3 at serine 10 (H3S10ph) has been associated with the increased expression of pro-inflammatory genes in colonic epithelial cells during colitis [125,126]. Dysregulation of histone deacetylases (HDACs) and histone acetyltransferases (HATs) directly impacts the acetylation state, shifting the balance toward increased HDAC expression and activity in IBD. This leads to HDAC-mediated transcriptional suppression of anti-inflammatory genes, thereby sustaining chronic inflammation [117].

Microbiota composition also influences histone modifications in IBD. The depletion of *Roseburia* spp. in UC patients correlates with reduced KHDC3L methylation and altered histone marks [127]. Additionally, Chen et al. [128] described an epigenetic mechanism whereby SETD8 regulates p62 expression, reducing the inflammatory response in colitis; p62 is elevated in IBD patients, suggesting that targeting SETD8 could be a promising therapeutic strategy. Together, these microbiota-driven and cell-intrinsic histone modifications ultimately affect T cell differentiation and cytokine production, contributing to the chronic inflammatory state.

#### 4.1.3. NcRNA in IBD

NcRNA is essential for the onset and progression of IBD. There exists a variation in the expression of ncRNAs between patients with IBD and controls, as well as between patients with CD and UC [129]. Based on its transcriptional properties and clinically significant features, lncRNA may be a useful biomarker for IBD [130]. Research has revealed that several lncRNAs, including H19, CDKN2B-AS1, GAS5, TUG1, CRNDE, and CARINH, are dysregulated in individuals with IBD, and these might act as useful biomarkers for diagnosis [60,131]. In patients with UC, the downregulation of the long non-coding RNA CDKN2B-AS1 which produces both linear and circularized RNA transcripts leads to improved colonic barrier integrity. This protective effect occurs through the suppression of Claudin-2 expression, a pore-forming tight junction protein that increases paracellular permeability [132]. In a separate study examining mRNA expression levels of various lncRNAs in colonic tissue and plasma samples from patients with IBD, including UC and CD, researchers found distinct expression patterns compared to healthy controls. These consistent changes across sample types highlight the potential of these lncRNAs as non-invasive biomarkers for IBD diagnosis and monitoring [133]. With the use of a mouse model of colitis and comprehensive bioinformatics analysis, IL1B, CXCL1, MMP1, and MMP10 were found to be markers of UC. Likewise, they hypothesized that the lncRNA XIST-miR-9-5p/miR-129-5p/miR-340-5p-NF-κB axis would have a competing endogenous RNA (ceRNA) network to regulate NF-κB expression [134]. Thus, by understanding the complicated roles that lncRNAs play in the pathophysiology of IBD, new diagnostic techniques and therapeutic targets may be developed for improved management of this complex gastrointestinal disease.

miRNA has a role in the pathophysiology of UC in IBD, regulating the immune system, the intestinal epithelial barrier, and the balance between the host and gut flora [126]. The gut microbiota profoundly influences miRNA expression patterns, and conversely, host-derived miRNAs shape microbial composition, creating a bidirectional regulatory axis that impacts disease pathogenesis. miRNA can affect immune cell maturation and differentiation in response to inflammation. For instance, miRNA-223 generated from bone marrow can alleviate mice colitis by suppressing NLRP3, which reduces the release of IL-1β [135]. The GIT is exposed to exosomes containing miRNA-155, which cause host macrophages to polarize toward M1, resulting in an increase in colitis [136]. Based on other research, active neutrophils may promote the production of pro-inflammatory molecules such miR-155 and miR-23a [137]. SCFAs can also promote miRNA expression in B cells and control B cell differentiation [44]. According to reports, colon samples from UC and CD patients had higher levels of miR-31. This protein reduces the production of the signaling protein GP130 as well as the inflammatory cytokine receptors Il7R and Il17RA, which in turn reduces inflammatory responses in epithelial cells [138]. Patients with UC and CRC were shown to have higher levels of miR-222-3p in their colons. By reducing miR-222-3p in IECs, oxidative damage decreased by activating Nrf2/HO-1 signaling through BRG1 targeting [139]. It has been established that miR-214 is related to the onset of UC and may increase inflammation; its absence decreased the severity of colitis. Ginsenoside Rh2 may act as a type of UC treatment by reducing the amount of STAT3/miR-214, according to a subsequent investigation [140]. According to Xu et al. [141], patients with UC had low expression of DNA methyltransferase DNMT3A and high expression of SMARCA5 and miR-182-5p. To be more specific, miR-182-5p may exacerbate UC by suppressing the Wnt/β-catenin signaling cascade through DNMT3A-mediated methylation of SMARCA5; conversely, miR-182-5p suppression may prevent UC by constraining HuR-mediated autophagy. Blood samples from UC patients showed a substantial elevation of miR-199a-5p when compared to their healthy counterparts [142]. Increased expression of p65, SMAD7, and AP1 due to decreased expression of miR-195-5p may account for the steroid resistance mechanisms in some UC patients [143]. In the colonic mucosa of UC and CD patients, miR-375-3p was downregulated. This meant that miR-375-3p-mediated elevation of TLR4 might activate NF-κB signaling, which in turn would increase the production of pro-inflammatory molecules [144].

Microbiota-induced miRNA dysregulation further illustrates this crosstalk. For example, activation of the Th17 pathway triggers the release of miR-223, which directly targets Claudin-8 (CLDN8), a key tight junction protein, thereby disrupting the intestinal epithelial barrier and increasing permeability [145]. In another mechanism, *Fusobacterium nucleatum* activates the TLR4/MYD88 signaling pathway, leading to the suppression of miR-18a and miR-4802. This reduces miRNA-mediated repression of their target genes (such as ULK1 and ATG7), ultimately promoting autophagy a process that can contribute to disease progression or altered cellular responses in the context of IBD or related conditions [146]. Conversely, the host can employ miRNAs to modulate the composition and growth of the gut microbiota. According to Liu et al., fecal miR-30d targets *Akkermansia muciniphila* and upregulates lactase expression in this bacterium, thereby increasing its abundance in the gut [147]. Additionally, certain miRNAs, including miR-199a-5p and miR-1226, can selectively regulate the proliferation of bacteria such as segmented filamentous bacteria (SFB) and *Fusobacterium nucleatum* [148]. Overall, the interaction between the intestinal microbiota and miRNAs is bidirectional: the microbiota influences miRNA expression, while miRNAs in turn shape microbial communities. This crosstalk contributes to changes in inflammatory processes and intestinal homeostasis in IBD [149]. Collectively, these findings illustrate how microbial dysbiosis (such as reduced SCFA producers and overgrowth of *F. nucleatum*) drives specific miRNA alterations that impair barrier function, promote autophagy, and sustain inflammation, thereby linking microbiota composition, epigenetic regulation, and immune dysfunction in a self-reinforcing loop. These findings underscore the intricate role of miRNAs in mediating host–microbiota interactions, providing novel insights into maintaining gut balance and potential therapeutic strategies for IBD.

In summary, IBD pathogenesis is driven by a triad of microbial dysbiosis (reduced SCFA producers and overgrowth of pathogenic bacteria), epigenetic alterations (aberrant DNA methylation, HDAC/HAT imbalance, and miRNA dysregulation), and immune dysfunction (impaired Treg function, Th17 skewing, and barrier defects). These interconnected mechanisms create a self-reinforcing loop that sustains chronic inflammation and tissue injury.

### 4.2. Rheumatoid Arthritis (RA)

RA is a chronic inflammatory autoimmune disease that is characterized by synovitis. Clinical manifestations of RA involve cartilage erosion, injury, and swelling of the joints, along with a continuous state of inflammation [150]. Although the etiology of RA is unknown, environmental and genetic variables have been shown to play a role in the disease’s progression [151]. Epigenetic alterations in RA have been studied on peripheral blood mononuclear cells (PBMCs) and different immune cell types, including monocytes, T cells, and B cells [152]. The majority of evidence on the function of epigenetics in RA disease comes from investigations on fibroblast-like synoviocytes (FLSs) and synovial tissue [153]. In an inflammatory environment, these cells develop an aggressive and proliferative character. They also produce a considerable quantity of cytokines and chemokines, which attract and activate additional immune cells, eventually becoming the principal agents in the destruction of bone and cartilage by upregulating matrix-degrading enzymes in the joint [154]. Emerging evidence positions the gut microbiota as a critical environmental factor that shapes these epigenetic landscapes and, together with immune dysregulation, drives RA pathogenesis (Table 3). Figure 2 shows the role of epigenetic dysregulation in *RA*.

#### 4.2.1. DNA Methylation in Immune Cells and FLSs in RA

Recent studies have demonstrated that RA patients’ T cells and monocytes had worldwide DNA hypomethylation in comparison to those of healthy individuals. DNA methylation alterations in B cells during the early stages of RA in patients who have not yet received treatment compared to healthy donors, were revealed using genome-wide analysis utilizing microarrays [155]. Studies have demonstrated that CpG methylation within the promoter region of the IL-6 gene influences the pathogenesis of rheumatoid arthritis (RA) in PBMCs from RA patients [156,157]. As the disease progresses, elevated cytokine levels are partly attributable to promoter demethylation at a single CpG site in both the IL-6 and IL-10 genes, which enhances their transcriptional activity and contributes to sustained inflammation [158]. Cribbs et al. explored the dysfunctional activity of regulatory T cells (Tregs) in RA patients [159]. They found that the CTLA-4 promoter, specifically at the 658 CpG site, exhibits hypermethylation in RA patients compared to healthy controls. This hypermethylation blocks the binding of the cytoplasmic isoform of nuclear factor of activated T cells (NF-ATc2), leading to reduced CTLA-4 expression. Consequently, Tregs fail to induce the tryptophan-catabolizing enzyme indoleamine 2,3-dioxygenase (IDO), impairing activation of the immunosuppressive kynurenine pathway and compromising Treg-mediated immune regulation.

Additionally, CD4^+^ T cells from RA patients display global hypomethylation. In particular, the IFNγ promoter in CD4^+^CD8^+^ T cells (a subset often expanded in RA) is markedly hypomethylated, resulting in enhanced production of IFNγ and promoting a pro-inflammatory Th1-like phenotype that exacerbates joint inflammation and tissue damage. These epigenetic alterations in key immune genes highlight how aberrant DNA methylation patterns in circulating immune cells contribute to dysregulated cytokine production, impaired Treg function, and amplified inflammatory responses central to RA pathogenesis. Targeting T cells could be a powerful strategy to uncover new treatment targets. A recent study found that the DNA methylation of T cells in RA patients differed dramatically between their synovium and blood. A recent study found that rats with RA have significantly lower levels of the secreted frizzled-related protein 2 (SFRP2). Overexpression of SFRP2 in FLS of RA rats reduces canonical Wnt signaling and slows RA development. Furthermore, FLSs in RA exhibit metabolic anomalies impacted by cytokines and activated T cells, which in the early stages of the disease contribute to inflammation and joint destruction [160]. Environmental factors, including alterations in gut microbiota composition, may contribute to these methylation patterns by influencing the availability of methyl donors (folate and methionine) and modulating one-carbon metabolism [151]. The gut microbiota likely contributes to these methylation changes through metabolite production; however, direct mechanistic studies in RA remain limited. Thus, further study is required to understand the interactions between microbiota, immune cells, FLSs and DNA methylation to develop target therapeutic strategies for RA.

#### 4.2.2. Extracellular Vesicles (EVs) in RA Pathogenesis

In recent years, extracellular vesicles (EVs) have emerged as critical mediators of intercellular communication in RA. These membrane-bound vesicles, including exosomes and microvesicles, carry a diverse cargo of proteins, lipids, nucleic acids (mRNAs, miRNAs and lncRNAs), and epigenetic modifiers that can be transferred between immune cells and FLSs, thereby shaping the inflammatory milieu [161].

EVs derived from RA patients exhibit potent immunomodulatory properties. Specifically, RA-derived exosomes have been shown to activate DCs, promoting their maturation and enhancing their capacity to prime T cell responses. Mechanistically, these EVs carry surface-expressed damage-associated molecular patterns (DAMPs) and enriched pro-inflammatory miRNAs that trigger TLR-mediated signaling in DCs, leading to increased production of IL-6, IL-1β, and TNF-α and driving Th17 differentiation [162]. This EV-mediated DC activation contributes to the breakdown of immune tolerance and amplifies the autoimmune cascade in RA.

Autophagy and PTMs play pivotal roles in EV biogenesis and their immunogenic cargo sorting. Autophagy-related proteins (such as LC3 and ATG5) regulate the formation of EV subtypes and influence the packaging of citrullinated peptides and pro-inflammatory cytokines into EVs. Notably, citrullinated proteins, which are hallmark autoantigens in RA, are enriched in RA synovial fluid EVs and are recognized by anti-citrullinated protein antibodies (ACPAs), forming immune complexes that further activate DCs and B cells. Post-translational modifications such as ubiquitination, SUMOylation, and phosphorylation of EV cargo proteins modulate their stability, sorting, and biological activity [161,162].

The gut microbiota has been implicated in shaping the EV landscape in RA. Bacterial-derived EVs (outer membrane vesicles) can translocate from the gut to the synovium, carrying microbial antigens, lipopolysaccharide, and metabolites that influence host immune responses. These microbial EVs have been shown to induce pro-inflammatory cytokine production in synovial fibroblasts and to modulate the epigenetic machinery of recipient cells, including histone modifications and miRNA expression [39]. The interplay between host-derived EVs, microbial EVs, and the gut–joint axis represents a novel frontier in understanding RA pathogenesis and may offer new therapeutic targets.

Collectively, these findings underscore that EVs are not merely passive shuttles but active participants in the immunopathogenesis of RA. Targeting EV biogenesis, cargo sorting, or uptake may provide innovative strategies to interrupt the vicious cycle of inflammation, dendritic cell activation, and autoimmunity.

#### 4.2.3. Histone Modifications in Immune Cells and FLSs in RA and FLSs

Recent investigations suggest that histone alterations may play a role in the genesis and progression of RA. Studies have demonstrated a link between increased inflammation and joint deterioration in RA and certain histone modifications, such as histone acetylation and methylation [163]. PBMCs from RA patients have decreased class I HDAC expression and activity, which is linked to disturbing the balance between HDAC and HAT activity [164]. The decreased expression and activity of HDACs results in a hyperacetylation state that exacerbates pro-inflammatory processes and eventually causes RA. Mice with a T cell-specific HDAC1 deficiency (HDAC1-cKO) had no change in the antibody response to type II collagen and were resistant to the development of collagen-induced arthritis (CIA) [165]. HDAC inhibitors have been demonstrated to improve the activity of regulatory T cells and decrease the synthesis of pro-inflammatory cytokines including TNF-α and IL-6, which may assist in suppressing the immunological response in RA [165,166]. HDAC-selective inhibitors prevented human CD4^+^ T cells and a murine model of elevated Th17 from upregulating the chemokine receptor 6 (CCR6) [165]. Several HDAC inhibitors, including HDAC6 inhibitors CKD-506, CKD-L [167] and HDAC6 inhibitor NKHDAC1 [168], are now being investigated as potential therapies for RA in preclinical research.

Direct studies of histone alterations in synovial fibroblasts are relatively infrequent. Notably, there are conflicting reports regarding *HDAC* expression and activity in RA synovial tissue. Huber et al. [169] found a decreased expression of *HDAC1* and *HDAC2*, resulting in overall hyperacetylation, suggesting a shift in the HAT/HDAC balance toward acetylation. In contrast, Kawabata et al. [170] reported increased HDAC1 expression and total HDAC activity in RA synovial tissue. These discrepancies may reflect differences in disease stage, cell type composition, or sample processing, highlighting the complexity of epigenetic regulation in RA and the need for cell type specific analyses to resolve these inconsistencies.

It was recently discovered that inhibiting *HDAC3* expression can lower pro-inflammatory factors in RASFs almost as well as general HDAC inhibition. This suggests that *HDAC3* could be a suitable target for specific therapy [171]. Collectively, these findings position HDAC as a promising therapeutic target for RA patients. Beyond their well-established role in the pathogenesis of CIA a widely used animal model of RA-HDAC inhibition has shown potential to modulate inflammatory pathways and joint damage in human disease.

Quantitative analyses, including immunohistochemistry and Western blotting, demonstrated a striking 13.8-fold to 15.5-fold increase in H3K79 methylation levels in the synovial tissue of RA patients compared to non-arthritic controls. This hypermethylation likely contributes to the sustained expression of pro-inflammatory genes and fibroblast-like synoviocyte activation, further driving synovial hyperplasia, pannus formation, and cartilage/bone destruction characteristic of RA [172]. These observations reinforce the importance of histone methylation machinery particularly DOT1L-mediated H3K79me in RA pathogenesis and highlight DOT1L as another potential epigenetic target for novel disease-modifying therapies. The presence of aberrant HKM patterns in RASF points to dysregulation of HKMTs and HKDMs in these cells.

Specifically, upon stimulation with TNF-α, the expression levels of four HKDMs—FBXL10 (also known as KDM2B), JMJD2D (KDM4D), NO66 (also known as MINA or RIOX2), and FBXL11 (KDM2A) which catalyze demethylation at histone H3 lysine residues (H3K4, H3K9, or H3K36) are significantly higher in RASF compared to osteoarthritis synovial fibroblasts (OASFs). By altering HKM profiles, these dysregulated HKM-modifying enzymes contribute to changes in gene expression within RASF, thereby influencing key processes in RA pathogenesis, such as persistent inflammation, synovial hyperplasia, and joint destruction [173]. The Jumonji C domain-containing histone demethylase JMJD3 (also part of the JMJD3/KDM6B family) plays a central role in regulating FLS proliferation and activation, processes closely linked to progressive joint deterioration and degenerative changes in RA [174]. JMJD3 expression is markedly elevated in RA-FLS compared to healthy or non-RA controls. In FLS, platelet-derived growth factor (PDGF), a potent mitogen implicated in RA induces this upregulation, which in turn promotes FLS migration and proliferation. Inhibiting JMJD3 activity substantially reduces PDGF-driven FLS proliferation and migration. Furthermore, the knockdown of JMJD3 abolishes PDGF-induced expression of proliferating cell nuclear antigen (PCNA), a key marker of cell proliferation, and attenuates the inflammatory responses of synovial fibroblasts when stimulated with interleukin-1β (IL-1β) [175]. These findings collectively illustrate how altered histone demethylase activity in synovial fibroblasts contributes to the aggressive, invasive phenotype of RASF/FLS in RA, highlighting JMJD3 and other HKDMs as potential epigenetic targets for therapeutic intervention to mitigate synovial inflammation and joint damage. Therefore, JMJD3 is crucial to the progression of RA. Hence, focusing on JMJD3 could be a novel approach to the identification and management of RA.

The gut microbiota has been found to influence immune responses and gut homeostasis via histone modifications, and this may extend to RA [43]. Gut microbiota-derived SCFAs, particularly butyrate and propionate, are known HDAC inhibitors that can suppress pro-inflammatory gene expression and promote Treg differentiation [36,127]. In RA, alterations in SCFA-producing bacteria (such as decreased Faecalibacterium and Roseburia) may contribute to the HDAC/HAT imbalance observed in immune cells and synoviocytes. Thus, in order to fully understand the intricate pathogenesis of RA and develop effective therapeutic approaches, further study is required to clarify the interactions between immune cells, FLSs, gut microbiota, and histone modifications.

#### 4.2.4. MicroRNA in Immune Cells and FLSs in RA

Patients with RA and different cells linked to RA have different levels of miRNA expression. For example, a variety of miRNAs were shown to be upregulated (miR-16, miR-103a, miR-132, miR-145, miR-146a, and miR-155) and downregulated (miR-21, miR-125b, and miR548a) in peripheral blood monocytes from individuals with RA; these may be connected with T cell homeostasis [176]. The aberrant expression of miR-17 and miR-146a was discovered to be connected with the imbalance of Treg cells in peripheral blood T cells [177]. Research has demonstrated that RA caused by particulate matter can generate ROS and trigger the MAPK signaling pathway, which in turn downregulates miR137 [178]. Thus, it is possible to hypothesize that focusing on miR137 may mediate ROS, offering novel approaches to the management of RA [163].

Researchers are increasingly focused on the gut microbiota as a key role in the onset and progression of RA. A study that entailed transplanting fecal samples from RA patients to GF mice indicated that the animals that received the microbiota of RA patients had higher numbers of Th17 cells in their stomachs and a higher risk of developing severe arthritis [179]. Preclinical RA mice showed an increase in *Firmicutes* and *Proteobacteria* (e.g., *Ruminococcaceae*, *Desulfovibrinocaceae*, and *Lachnospiraceae*,) and a decrease in *Bacteroidete* (e.g., S24-7 and *Bacteroidaceae*). Eliminating the gut microbiota in established RA animals decreased the number of T helper 17 cells that produced IL-17 and the severity of RA [180]. Significant variations in the composition of gut bacteria were found in a clinical investigation comprising 30 healthy controls and 32 RA patients. RA patients had significantly lower levels of *lactobacteria* (P < 0.05), but significantly higher levels of *enterococci* and *clostridia* (P < 0.05) were observed. The proportion of *Bifidobacteria*, *Bacteroids*, and *Lactopositive Colibacteria* decreased (P < 0.05), but opportunistic *Enterobacteria* and *Staphylococci* increased (P < 0.05) [181]. Notably, opportunistic *Enterobacteriaceae* were also found in the nasal mucosa and urine (P < 0.05). This finding suggested that *enterobacteriaceae* that feed on opportunities may move from the intestines to other organs [181]. These miRNA changes are intimately linked to gut microbiota composition. For example, particulate matter-induced RA involves downregulation of miR-137 via MAPK signaling [178], and miR-17/miR-146a imbalances are linked to Treg dysfunction [177]. Throughout the presentation of undifferentiated arthritis (UA)/RA, there may be a bidirectional regulation mechanism between the intestinal microbiota and miRNA, and an individual’s prognosis for UA may be influenced by their gut microbiome [182]. Through the integration of microbiota and miRNA investigations, researchers have the potential to identify novel biomarkers for more effective disease management and uncover critical mechanisms underlying the pathogenesis of RA.

Collectively, the pathogenesis of RA is driven by a complex interplay between gut microbiota dysbiosis (reduced SCFA producers and overgrowth of pathobionts), epigenetic alterations (DNA hypomethylation, HDAC/HAT imbalance, histone methylation changes, and miRNA dysregulation), and immune dysregulation (Th17 skewing, Treg dysfunction, FLS activation, and EV-mediated crosstalk). These interconnected mechanisms create a self-reinforcing inflammatory circuit that sustains synovitis and joint destruction. Understanding these links offers new opportunities for biomarker discovery and therapeutic intervention targeting the microbiota–epigenetics–immune axis.

### 4.3. Systemic Lupus Erythematosus (SLE)

SLE represents a classic systemic autoimmune disorder in which the aberrant production of antinuclear antibodies (ANAs), the breakdown of immune complexes in the affected organism, and a persistent immunological response [183]. This systemic disease is known as aberrant B cell compartment disease because hyperactivated B cells produce erroneous overstimulation of other immunological elements such as innate and adaptive immune cells such as dendritic cells or T cells. In lupus, B cells overproduce autoantibodies, resulting in an unregulated cycle of autoaggressive processes. The depletion of autoagressive B cells could be a useful treatment for autoantibody-driven diseases [184]. Although the precise etiology of SLE remains elusive, the disease is widely regarded as multifactorial, arising from complex interactions among multiple genetic susceptibility loci, epigenetic modifications, and environmental triggers. These intertwined genetic, epigenetic, hormonal, and environmental elements collectively drive the loss of immune tolerance, aberrant autoantibody production, and chronic inflammation characteristic of SLE, with the female bias reflecting a convergence of sex-specific immunological and epigenetic vulnerabilities. Figure 2 shows the role of epigenetic dysregulation in SLE.

#### 4.3.1. DNA Methylation in SLE

Epigenetic changes in DNA methylation play a pivotal role in regulating gene expression and contribute significantly to the pathogenesis of SLE. In SLE patients, an elevated expression of MBD2 and DNMT1 has been observed, potentially leading to aberrant DNA hypermethylation in certain contexts and subsequent dysregulation of gene expression [185]. Multiple studies examining CpG methylation patterns across various immune cell populations including CD4^+^ T cells, CD19^+^ B cells, and CD14^+^ monocytes from SLE patients have consistently identified global DNA hypomethylation particularly in CD4^+^ T cells, which is thought to promote the activation of autoreactive pathways [186]. A well-characterized example involves the integrin CD11a/CD18 (also known as LFA-1 or ITGAL), which mediates leukocyte adhesion and migration into inflamed tissues. Overexpression of CD11a/CD18 on T cells is strongly associated with the development of T cell autoreactivity in SLE [187]. Notably, adoptive transfer of T cells engineered to overexpress CD11a/CD18 into syngeneic mice induces a lupus-like autoimmune syndrome [188]. In patients with active SLE, the promoter region of CD11a/CD18 exhibits significant DNA hypomethylation in T lymphocytes, which directly correlates with increased gene expression and contributes to disease activity [189]. Similar epigenetic dysregulation affects other key immune genes. For instance, TNFSF7 promoter demethylation and consequent overexpression in SLE T cells. This leads to enhanced CD70 surface expression, which in turn promotes B cell activation and drives increased IgG production and autoantibody synthesis [190]. In female SLE patients, the CD40LG promoter shows DNA hypomethylation, resulting in its overexpression on CD4^+^ T cells a phenomenon linked to the X-chromosome location of CD40LG and potential escape from X-inactivation [191]. Collectively, these findings illustrate how locus-specific DNA hypomethylation in SLE immune cells particularly CD4^+^ T cells leads to inappropriate overexpression of pro-inflammatory and autoreactive molecules (CD11a/CD18, CD70, and CD40LG), thereby amplifying B cell hyperactivity, autoantibody production, and systemic inflammation central to SLE pathogenesis.

A thorough examination of the DNA methylation patterns in CD4^+^ T cells from lupus patients and healthy controls indicated that out of 27,578 CG sites discovered in the promoter regions of 14,495 genes, 105 were hypermethylated and 236 were hypomethylated [192]. A study on the DNA methylome in naïve CD4^+^ T lymphocytes in lupus included two groups of patients and healthy controls. This study looked at genome-wide DNA methylation. To investigate the relationship between observed changes in DNA methylation and levels of mRNA expression, the study quantified over 485,000 methylation sites across the genome and identified and replicated 86 differentially methylated CG sites between patients and controls in 47 genes, the majority of which were hypomethylated based on gene expression analysis from the same cells. Hypomethylation in naïve T cells from lupus patients disrupts regulated genes, including IFIT1, IFIT3, MX1, STAT1, IFI44L, USP18, TRIM22, and BST2, indicating epigenetic transcriptional accessibility in these genomic locations [193]. Additionally, it has been shown that the pathophysiology of SLE is influenced by the hypermethylation of anti-inflammatory genes. Patients with SLE have higher serum and tissue levels of IL-10, which promotes B cells and leads B cells to produce autoantibodies [194]. In SLE, T cells exhibit elevated expression of IL-10, which is largely attributable to DNA hypomethylation within the IL-10 gene promoter. This epigenetic change facilitates the recruitment and binding of STAT3 to the IL-10 promoter complex, thereby enhancing transcriptional activation and driving increased IL-10 production. The resulting overexpression of IL-10 in SLE T cells contributes to immune dysregulation, as IL-10 while generally immunosuppressive can paradoxically promote B cell hyperactivity, autoantibody production, and sustained inflammation in the context of SLE. Together, these mechanisms, epigenetically driven IL-10 overexpression in T cells and IL-17A-mediated recruitment and amplification of innate immune responses, highlight the complex, dual nature of cytokine dysregulation in SLE, where both anti- and pro-inflammatory pathways are aberrantly activated and contribute to disease pathogenesis. SLE development is related to IL-17A [195]. The IL-17A promoter has been associated with the highly expressed cAMP-responsive element modulator α (CREMα), which causes SLE T cells to produce more IL-17A. Table 3 displays a number of significant methylation alterations in SLE conditions.

Unbalanced gut microbiota has been associated with immunological responses and inflammation in SLE patients, which may have an impact on DNA methylation and other epigenetic modifications. In particular, certain microbial metabolites may influence the methylation machinery of the host, leading to modifications in the expression of genes linked to autoimmune processes [196]. Furthermore, research has shown a correlation between particular bacterial taxa and distinct DNA methylation profiles, indicating a potential function for the gut microbiome in regulating epigenetic modifications that contribute to the pathogenesis of SLE [197]. The exact methods, however, are still unknown because some research suggests that although microbiota can influence methylation, the opposite may also be true, which makes sense of this bidirectional link difficult to explain [198]. Furthermore, the variations in results between other groups highlight the necessity for more investigation to better understand these relationships and their consequences for the management of SLE.

**Table 2:** Comparative overview of major epigenetic alterations in IBD, RA, and SLE.

Epigenetic Mechanism	Inflammatory Bowel Disease (IBD)	Rheumatoid Arthritis (RA)	Systemic Lupus Erythematosus (SLE)	Ref.
**DNA methylation**	Global hypomethylation; promoter hypermethylation of *IL-10*, tight junction genes; hypomethylation of *RPS6KA2*	Global hypomethylation in PBMCs, T cells, monocytes; promoter hypomethylation of *IL-6*, *IL-10*, *IFNγ*; hypermethylation of *CTLA-4* in Tregs	Global hypomethylation in CD4^+^ T cells; hypomethylation of *CD11a*, *CD70*, *CD40LG*, *IL-10*, *IL-17A*; hypermethylation of *FOXP3*, *IL-2*	[117,123,156,159,186,189,193]
**Histone modifications**	Increased H3S10ph; altered HDAC/HAT balance; changes in H3K4me3, H3K27me3	Decreased class I HDAC activity in PBMCs; hyperacetylation in synovial tissue; increased H3K79me; dysregulated JMJD3, H3K4me3, H3K9me3	Global hypoacetylation in CD4^+^ T cells; altered H3K4me3 at *WDR5*, *PTPN22*; increased H3K27me3 at *HPK1*; CREMα-mediated deacetylation at *IL-2*	[125,126,164,171,199]
**MicroRNAs**	Dysregulated miR-31, miR-222-3p, miR-214, miR-182-5p, miR-375-3p; miR-223 targets *CLDN8*; microbiota-miRNA crosstalk (miR-30d, miR-199a-5p)	Upregulated miR-16, miR-146a, miR-155; downregulated miR-21, miR-125b, miR-137; miR-17, miR-146a linked to Treg imbalance; miR-137 mediates ROS signaling	Upregulated miR-21, miR-34a, miR-152-3p; downregulated miR-17-5p, miR-146a, miR-155; miR-10b-5p targets *SRSF1*; miR-125a targets *KLF13*; miR-142-3p increases pro-inflammatory cytokines	[138,145,176,178]

**Table 3:** DNA methylation status, influences/effects of aberrant methylation, and types of studies/cells analyzed for DNA methylation assessment in SLE-associated genes.

Genes Type	Cell Type/Studies (In Vitro)	DNA Methylation Level	Effect in SLE	References
IL-2	CD3^+^ T cells, CD4^+^ T cells, Effector CD4^+^ T cells	**	Treg suppression	[199,200]
IL-10	T cells	*	Activation of the B cell	[194]
IL-17A	T cells, CD4^+^ T cells, B cells	*	A reunion of neutrophils and monocytes	[201]
CD11a/CD18	CD4^+^ T cells	*	Leukocyte migration and adhesion in inflammatory tissues	[202,203]
CDKN1A	PBMCs	**	Potential effects on apoptosis and DNA repair	[200]
SOCS1	CD4^+^ T	**	Immune dysregulation	[200]
TREX1	PBMCs	**	Impaired exonuclease activity, cytosolic DNA accumulation, and autoreactive cell survival	[204]
CD70	CD4^+^ T cells	*	B cell activation	[203]
CD40LG	CD4^+^ T cells	*	B cell maturation	[191]
MX1, BST2, STAT1, TRIM22, IFIT1, IFIT3, IFI44L, USP18	naïve CD4^+^ T cells	*	Type I IFN-mediated responses	[193]
FOXP3	Treg	*	Decreased number and modified functionality of regulatory T cells	[205]
PARP9, DT3XL, IFI44, RSAD2, PLSCR1, IRF7	PBMCs	*	Type I IFN-mediated responses	[206]
BCL6	naïve CD4^+^ T cells	*	Tfh cell differentiation	[207]
CREM	CD3^+^ T cells, CD4^+^ T cells, Effector CD4^+^ T cells	*	Responsible for DN T cell production as well as CD4^+^ T cell IL-2 and IL-17 regulation	[205]

Note: * Reduced DNA methylation; ** increased DNA methylation.

#### 4.3.2. Histone Modifications in SLE

Histone alterations have a significant impact on the pathogenesis of systemic SLE, influencing gene expression and immune responses. Research suggests that specific histone modifications, such as acetylation and methylation, are altered in SLE patients, contributing to the deregulation of immune-related genes and triggering autoimmunity [208]. In SLE, CD4^+^ T cells from patients exhibit reduced global histone acetylation as well as decreased H3K9 methylation compared to healthy controls. Genome-wide analyses of histone modifications in PBMCs from SLE patients have revealed locus-specific alterations in H3K4me3. Specifically, H3K4me3 levels are significantly increased at the genes encoding WD repeat-containing protein 5 (WDR5) a core component of histone methyltransferase complexes and solute carrier family 24 member 3 (SLC24A3), potentially enhancing their expression and contributing to dysregulated cellular processes [209]. Conversely, H3K4me3 levels are decreased at several other genes, including protein tyrosine phosphatase non-receptor type 22 (PTPN22) (a key negative regulator of T cell signaling), methyltransferase-like 16 (METTL16) (involved in RNA methylation), LDL receptor-related protein 1B (LRP1B), and cadherin 13 (CDH13). In SLE CD4^+^ T cells, another notable change involves elevated H3K27me3 at the promoter and/or gene body of hematopoietic progenitor kinase 1 (HPK1). This increase in repressive H3K27me3 leads to downregulation of the HPK1 expression. Since HPK1 normally functions as a negative regulator of T cell receptor signaling and activation, its reduced levels promote excessive T cell activation, proliferation, and cytokine production key drivers of the autoimmune pathology in SLE [210]. These findings collectively illustrate how dysregulated histone modifications, ranging from global hypoacetylation and hypomethylation to gene-specific gains or losses of active H3K4me3 and repressive H3K27me3 marks, contribute to the aberrant transcriptional landscape in SLE immune cells, particularly CD4^+^ T cells, thereby sustaining chronic inflammation and autoreactivity. In SLE CD4^+^ T cells, decreased JMJD3 binding leads to higher H3K27me3 enrichment in the HPK1 promoter. CD4^+^ T cells from active SLE patients demonstrated widespread hypoacetylation of histones H3 and H4 [211]. Evidence suggests that di-methylation of lys4 H3 increases the promoter of the TNFSF7 (CD70) gene in SLE CD4^+^ cells, which is linked to disease activity.

RFX1 acts as a transcriptional repressor in SLE by recruiting HDAC1 (histone deacetylase 1) to reduce histone H3 acetylation (H3ac) levels and SUV39H1 (a histone methyltransferase) to increase repressive H3K9me3 marks, thereby silencing target genes involved in immune regulation [212]. In SLE CD4^+^ T cells, overexpression of CREMα (cAMP-responsive element modulator α) suppresses IL-2 production through multiple epigenetic mechanisms: it promotes deacetylation of H3K18 (via HDAC recruitment) and induces DNA hypermethylation at the IL-2 promoter through the concerted action of HDAC1 and DNMT3A (DNA methyltransferase 3A) [199]. Treatment of SLE CD4^+^ T cells with Trichostatin A (TSA), a broad-spectrum HDAC inhibitor, reverses some of these dysregulations: it decreases the expression of CD40LG and IL-10 genes while increasing IFNγ gene expression, highlighting the therapeutic potential of targeting HDAC activity to restore balanced cytokine profiles [213]. Activation of TLR2 in SLE CD4^+^ T cells drives a pro-inflammatory phenotype by upregulating expression of CD40LG, CD70, IL-6, IL-17A, IL-17F, and TNFα, while simultaneously downregulating FOXP3, the master transcription factor for Treg differentiation and function. At the epigenetic level, TLR2 signaling increases H4 acetylation (H4ac) and decreases repressive H3K9me3 at the promoters of IL-17A and IL-17F, resulting in enhanced transcription of these Th17-associated cytokines.

In SLE CD4^+^ T cells, reduced H3K4me3 at the promoter of TNFAIP3, key negative regulator of NF-κB signaling leads to decreased TNFAIP3 expression. This downregulation impairs negative feedback on inflammatory pathways, resulting in elevated production of IFNγ and IL-17 and contributing to sustained autoimmunity [214]. Furthermore, BCL6 (B cell lymphoma 6) is markedly upregulated in SLE CD4^+^ T cells, where it represses expression of miR-142-3p and miR-142-5p by enhancing repressive H3K27me3 and reducing permissive H3K9/K14 acetylation at the miR-142 promoter. BCL6 recruits EZH2 (the catalytic subunit of Polycomb repressive complex 2, responsible for H3K27me3) and HDAC5 to the promoter region. This epigenetic silencing of miR-142 promotes the expression of its downstream targets, including IL-21 and ICOS (inducible T cell costimulator), which are critical for T follicular helper (Tfh) cell function, germinal center formation, and autoantibody production in SLE [215]. These examples collectively demonstrate how complex, interconnected epigenetic alterations spanning histone acetylation/deacetylation, site-specific methylation/demethylation, DNA methylation, and non-coding RNA regulation in SLE CD4^+^ T cells drive cytokine imbalance, loss of Treg function, Th17/Tfh skewing, and persistent autoreactivity, offering multiple potential targets for epigenetic-based therapies. BCL6 attracted EZH2 and HDAC5 to the miR-142-3p/5p promoter, where they encouraged the synthesis of IL-21, ICOS, and CD40LG. BCL-6, IL-21, ICOS, and CD40LG are all required for the creation and function of Tfh cells, which leads to CD4^+^ T cell hyperactivity and autoantibody production in SLE. IL-23 increased the phosphorylation of STAT3 in SLE patients’ Th17 cells, which are CD4^+^ T helper cells that generate IL-17 [216]. IL-23 elevated H3K4me3 levels and decreased H3K27me3 levels in the RORγt gene, which is a major regulator of Th17 cells. IL-23-induced STAT3 interacts to the RORγt gene locus, boosting its expression [217]. There are multiple important histone alterations in SLE patients, as seen in (Table 4).

Studies have shown a link between SLE and GM metabolites such as lipids, amino acids, and SCFAs. SCFAs decrease histone deacetylase activity and stimulate Treg development, which secretes the anti-inflammatory cytokine IL-10 [41]. It has been shown that GM dysbiosis in SLE is associated with altered SCFA production, as evidenced by elevated fecal SCFA levels and reduced *Firmicutes*/*Bacteroidetes* F/B ratio [218]. Sodium valproate, a SCFA, has been shown to activate monocyte-derived macrophages in an alternative manner in SLE patients, hence promoting the anti-inflammatory immune response [219]. Additionally, a study of blood samples from SLE patients following a fecal microbiota transplantation (FMT) showed a decrease in IL-6 levels and a decrease in the CD4^+^ memory/naïve ratio, as well as an increase in GM-derived SCFAs [220]. However, further research is necessary to completely understand the intricate relationships between microbiota and histone modifications in SLE, as the precise mechanisms by which these interactions occur are still unclear.

**Table 4:** This table lists the states of histone modifications, their effects, and the different kinds of experiments and cell types that were used to evaluate histone modifications in SLE-associated genes.

Genes Type	Cell Type/Experiments (In Vitro)	Modification	Effect in SLE	References
IL-2	CD3^+^ T cells, CD4^+^ T cells, effector CD4^+^ T cells	H3K18 deacetylation, H3K27 trimethylation	Decreased activation-induced cell death, prolonged autoreactive T cell survival, and compromised generation of regulatory T cells; impaired cytotoxic CD8^+^ T cell activity, effector CD4^+^ T cell differentiation, and cytokine expression	[195,199]
IL-17	T cells	high H3ac	The collection of neutrophils and monocytes	[221]
IL-17A, IL-17F	CD4^+^ T cells	high H4ac, low H3K9me3	The collection of neutrophils and monocytes	[222]
CD11a/CD18	CD4^+^ T cells	low H3K9me3, high H3ac	leukocyte adhesion and migration in inflamed tissues	[212]
CD8A, CD8B	CD4^+^ T cells, CD8^+^ T cells, DN T cells/in vitro	H3K18 deacetylation, H3K27 trimethylation in DN T cells	Generation of CD3^+^ CD4^−^ CD8^−^ DN T cells	[223]
CD70	CD4^+^ T cells	high H3K4me2, high H3ac	Activation of the B cell	[224]
CD70	CD4^+^ T cells	low H3K9me3, high H3ac	T cell activation	[212]
TNFα	Monocytes	high H4ac	Inflammatory responses	[225]
ITGAL	CD4^+^ T cells	Reduced H3K27 trimethylation through histone demethylase	Increased T cell-mediated inflammation	[226]
RORγt	CD4^+^ T cells	high H3K4me3, low H3K27me3	Th17 differentiation	[216]
TNFAIP3	CD4^+^ T cells	low H3K4me3	production of IFNγ and IL-17	[214]

#### 4.3.3. MicroRNAs in SLE

miRNAs play a significant role in the pathogenesis of SLE, influencing various immune responses and disease manifestations. In SLE T cells, estrogen-induced miR-10b-5p is increased. SRSF1 is the target of miR-10b-5p, as it promotes the synthesis of IL-2. As a result, in SLE, the reduction in SRSF1 impairs Treg development and causes autoreactive T cells to persist [227]. In SLE, B cells expression of miR-17-5p is suppressed by the transcription factor E2F2 (E2F transcription factor 2) [228]. miR-17-5p directly targets and negatively regulates IL-10, a cytokine that promotes B cell activation, survival, and differentiation. By inhibiting miR-17-5p, E2F2 relieves this repression, leading to increased IL-10 levels that drive excessive B cell stimulation and contribute to heightened autoantibody production a hallmark of SLE pathogenesis. In contrast, miR-21 expression is significantly elevated in CD4^+^ T cells from SLE patients, and its levels correlate positively with disease activity as measured by the SLEDAI (Systemic Lupus Erythematosus Disease Activity Index) score [229]. This upregulation of miR-21 likely amplifies pro-inflammatory signaling and T cell dysfunction in active disease. Furthermore, pro-inflammatory cytokines such as TNFα and IL-6—which are commonly elevated in SLE—activate the NF-κB/p65 pathway in PBMCs and CD4^+^ T cells. Activated NF-κB/p65 translocates to the nucleus and binds directly to the promoter region of miR-34a, thereby enhancing its transcription and increasing miR-34a expression [relevant studies]. Elevated miR-34a, in turn, may modulate downstream targets involved in apoptosis, cell cycle regulation, and immune tolerance, further perpetuating the inflammatory and autoreactive environment characteristic of SLE [230]. These findings highlight the complex, cell-type-specific roles of microRNAs in SLE: miR-17-5p downregulation in B cells promotes humoral autoimmunity via IL-10 derepression, while miR-21 and miR-34a upregulation in T cells correlates with disease activity and sustains T cell-mediated inflammation. Together, these miRNA dysregulations represent promising epigenetic biomarkers and potential therapeutic targets in SLE. miR-34a targets FOXP3, which stimulates the development of Treg cells. Certain miRNAs (miR-189, miR-61, miR-78, miR-21, miR-142-3p, miR-342, miR-299-3p, miR-198, and miR-298) increased in peripheral blood mononuclear cells from SLE patients, but others (miR-196a, miR-17-5p, miR-409-3p, miR-141, miR-383, miR-112, and miR 184) declined [231]. miR-125a targets Kruppellike factor 13 (KLF13), which upregulates RANTES expression. In SLE monocyte-derived DCs, miR-142-3p expression rises, resulting in increased production of CCL2, CCL5, CXCL8, IL-6, and TNFα [232]. Further studies identified STAT1 as an additional target of miR-146a, revealing an inverse relationship between miR-146a expression levels and both the induction of interferon-inducible genes and SLE disease activity [233]. In patients with SLE, elevated expression of miR-152-3p is associated with key clinical manifestations, including skin rashes, arthritis, positivity for anti-double-stranded DNA (anti-dsDNA) antibodies, and elevated serum IgG levels. Mechanistically, miR-152-3p promotes CD4^+^ T cell autoreactivity and enhances TLR-mediated inflammatory responses by directly targeting and suppressing DNMT1 (DNA methyltransferase 1). This repression of DNMT1 leads to reduced DNA methylation at the MyD88 gene promoter, resulting in increased MyD88 expression and subsequent amplification of Toll-like receptor signaling, which drives pro-inflammatory cytokine production and contributes to SLE pathology. In contrast, miR-155 expression is significantly reduced in the serum, urine, and PBMCs of SLE patients [234]. miR-155 normally targets the transcription factor CREB (cAMP response element-binding protein), thereby limiting the expression of protein phosphatase 2A (PP2A). In SLE, downregulation of miR-155 relieves this repression, leading to increased PP2A levels. Both miR-155 deficiency and elevated PP2A contribute to suppressed IL-2 production in T cells, further impairing T cell function and immune regulation.

Moreover, miR-155 expression in SLE patients shows an inverse correlation with disease activity as measured by the SLEDAI score and with the presence of proteinuria, indicating that lower miR-155 levels are linked to more severe disease. Conversely, miR-155 expression is positively correlated with WBCs, suggesting a protective role in maintaining leukocyte homeostasis [235]. These findings underscore the dual, context-dependent roles of specific microRNAs in SLE: miR-152-3p acts as a disease-promoting factor by disrupting DNA methylation and amplifying innate immune signaling, while reduced miR-155 contributes to T cell dysfunction, IL-2 deficiency, and overall disease severity. Together, these miRNA alterations represent potential biomarkers for monitoring SLE activity and novel targets for epigenetic-based therapeutic strategies. Table 5 displays a number of significant miRNAs alterations in SLE patients. As a result, miRNAs are a promising field of research in SLE, with implications for understanding the disease and developing new therapeutics. Studies demonstrate that dysbiosis in the microbiota might lead to the generation of miRNAs that influence T cell differentiation and autoantibody synthesis, which are essential features of SLE [236]. However, the exact mechanisms by which microbiota dysbiosis influences miRNA regulation and, as a result, SLE the development remain unknown, emphasizing the need for additional study to explain these interactions and their implications for therapeutic options.

## 5. Potential Therapeutics

Studies on microbiome–host crosstalk and epigenetic dysregulation have enhanced our understanding of the pathogenic function and consequences for autoimmune and inflammatory diseases. Since microbial dysbiosis and epigenetic modifications are treatable with medication, they are a feasible strategy for targeted therapy. Study indicates that both DNA and RNA methylation play critical roles in the pathogenesis of conditions like SLE and RA [247]. In arthritic models, for example, DNA hypomethylating agents like decitabine have shown anti-inflammatory effects through the modulation of important transcription factors like interferon regulatory factor 8 (Irf8), which in turn restores immunological homeostasis [248]. Extensive research on epigenetic regulators has highlighted significant interest in HDAC inhibitors. These compounds work by inhibiting HDAC enzymes, which increases acetylation levels on lysine residues of both histones and non-histone proteins. This modification reduces the compact wrapping of DNA around histones, loosening chromatin structure and thereby enhancing accessibility for gene transcription factors [249]. In animal models of RA, the HDAC inhibitor TSA (trichostatin A) suppresses the production of pro-inflammatory cytokines by inflammatory synovial macrophages. Advances in understanding epigenetic mechanisms and improved experimental techniques have enabled the identification of various inhibitors targeting histone methyltransferases and demethylases. For example, inhibitors of lysine-specific demethylase 1 (LSD1/KDM1A) a FAD-dependent enzyme that demethylates H3K4 and H3K9 represent the only class of small-molecule histone demethylase inhibitors currently approved for clinical use [250]. Among these, chalcone-based LSD1 inhibitors (α,β-unsaturated aromatic ketones) show promising therapeutic potential across multiple conditions. This stems from their broad pharmacological effects, including antiulcer, antioxidant, anticancer, antiviral, anti-angiogenic, antibacterial, and notably anti-inflammatory activities [251]. Furthermore, clinical trials including numerous targets are also being carried out to develop and test miRNA inhibitors and mimetics. Serum concentrations of miRNA-5196 may serve as a biomarker for positive treatment results in persons with ankylosing spondylitis and RA treated with TNF-α [252]. MALAT1 has also been demonstrated to suppress miRNAs related in inflammation, such as miR155 and miR-125b. MiRNA targets are involved in multiple signaling pathways, such as NF-κB, JAK, STAT, p38, and JNK [253].

Live beneficial microbes are referred to as probiotics, whereas dietary fibers that promote the growth of these bacteria are called prebiotics. Both can impact the composition of the microbiome and may influence epigenetic alterations. The gut microbiota’s production of SCFAs is significant in circumstances like inflammation because it has the ability to influence the expression of genes associated with metabolism and inflammation via epigenetic mechanisms like DNA methylation and histone modifications [254], and promote anti-inflammatory pathways. Targeting epigenetic regulators, including NLRP3 and NF-κB inflammasome, can reduce inflammation in IBD models. Studies are being undertaken to determine the efficacy of specific probiotic strains in treating disorders like RA and IBD. Furthermore, nutritional interventions that change the composition of the gut microbiota can improve host–microbe interactions and possibly lower the severity of disorders like CD. Certain elimination or anti-inflammatory diets, such as the Mediterranean diet, may assist to alter the microbiota and minimize epigenetic dysregulation. Preliminary research indicates potential for restoring normal epigenetic functioning by microbiome reconstitution in autoimmune conditions such as IBD and Clostridium difficile infection [255]. Physical activity, stress management, and sleep hygiene can all influence both the epigenetic processes and microbiome. Studies on the specificity of FMT effects on microbe-host epigenetic interactions and dietary therapies are underway for a variety of autoimmune diseases, including lupus.

## 6. Challenges and Future Perspectives

The microbiome is extremely diverse and varied; this variety makes it more difficult to understand the way specific bacteria species interact with host epigenetic systems. Understanding the impact of microbial metabolites on the intricate biochemical processes involved in epigenetic control in host cells is an important challenge. Causative relationships between particular bacteria and epigenetic modifications are still difficult for researchers to pin down. Because food, lifestyle, and other environmental factors can alter the dynamic microbiome [256], it can be challenging to understand the association between these temporal changes in microbial composition and the epigenetic alterations that occur in host tissues over time. The full spectrum of epigenetic modifications and their functional consequences related to microbial impacts may not be accurately represented by current epigenomic sequencing and analysis approaches due to their resolution limitations.

A particularly promising avenue is the development of epigenetic biomarkers for autoimmune diseases. Cell-type-specific DNA methylation signatures [186,189,192,193], histone modification patterns [208,210], and circulating microRNA profiles [176,177,233] have the potential to serve as non-invasive diagnostic, prognostic, and treatment-response biomarkers. For instance, differentially methylated regions in CD4^+^ T cells have been associated with SLE disease activity [186,193], and specific miRNA panels are being explored for early RA detection [176,178]. However, validation in large, well-characterized cohorts and standardization of analytical pipelines are needed before clinical implementation.

Our understanding of the links between the microbiome and the host as well as the function of epigenetic modifications in disease processes will be enhanced by integrative studies combining microbiology, immunology, epigenetics, and bioinformatics. A key future direction is multi-omics integration, which involves the simultaneous analysis of metagenomics, epigenomics (DNA methylation, histone modifications, and non-coding RNAs), transcriptomics, proteomics, and metabolomics from the same individuals [87,109,123,124]. Such integrated approaches can reveal causal relationships between specific microbial taxa, their metabolites, and the resulting epigenetic and transcriptional changes in host immune cells. Machine learning models that incorporate multi-omics data may enable the identification of robust predictive signatures for disease onset, flare, and therapeutic response.

Research on the microbiota and epigenetics can help develop personalized therapeutic strategies for autoimmune and inflammatory disorders. Developing personalized therapies according to an individual’s epigenetic markers and microbiota profile has tremendous potential. The identification of microbial metabolites that promote advantageous epigenetic modifications may result in the development of probiotic or prebiotic treatments that target the epigenetic dysregulation associated with various diseases. In addition, effective translation of fundamental research findings to clinical settings is needed with particular focus on the ways in which epigenetic insights might guide autoimmune disease prevention, diagnosis, and therapy approaches.

## 7. Conclusions

In conclusion, the intricate link between the microbiome and host epigenetics highlights an important gap in our understanding of inflammatory and autoimmune diseases. This review highlights the significant impact that epigenetic dysregulation can have on the immune system, as influenced by microbial populations and their metabolites. As scientists continue investigating the complexity of these relationships, it becomes increasingly clear that restoring microbial balance may serve as a strong therapeutic strategy. Future research in this area not only intends to clarify the exact pathways via which dysbiosis influences epigenetic regulation but also seeks to build innovative microbiome-based therapies. Precision medicine techniques that improve host resilience and reduce the consequences of chronic inflammatory disorders can be advanced by incorporating insights from studies on microbiome–host interactions and epigenetic regulation.

## Figures and Tables

**Table 5:** Impact of miRNA expression changes, functional roles, types of research, and cells/samples used for miRNA expression assessment in SLE-associated genes/pathways.

Genes Type	Cell Type/Experiments (In Vitro)	miRNA Expression	Effect in SLE	References
IL-6	CD4^+^ T cells	miR-let-7a UP	IL-6 induction	[237]
SRSF1	T cells	miR-10b-5p up	Treg suppression	[238]
Blimp1, SOCS1	T cells	miR-let-7c Up	Activation of DCs	[239]
IL-10	B cells	miR-17-5p down	Autoantibody production	[228]
PDCD4	CD4^+^ T cells	miR-21 up	cell proliferation and B cell maturation	[229]
TAB2, TAB3, IKKα	T cells	miR-23b down	IL-17, TNF-α, IL-1β signaling	[240]
KLF13	T cells	miR-125a down	CCL5 induction	[241]
FOXP3	PBMCs, CD4^+^ T cells	miR-34a up	Treg suppression	[230]
FAS	CD4^+^ T cells	miR-98 down	apoptosis	[242]
EIF4EBP1	PBMCs	miR-99a-3p down	autophagy	[243]
TRAF6, IRAK1, TRAF6, IRAK1, IRAK2, IRF5, STAT1	CD4^+^ T cells	miR-146a down	Inflammatory response mediated by NFκB Type I IFN induction and signaling via the RIG-I-dependent antiviral pathway	[210,233]
KLF5	B cells	miR-152-3p up	increase in BAFF expression	[244]
CREB	PBMCs	miR-155 down	reduced IL-2 production	[234]
IRF9	monocytes	miR-302d down	type I IFN-mediated responses	[235]
TGF-β1	BMSCs	miR-663 up	Activation of Tfh cell and Treg suppression	[245]
TLR	monocytes, macrophages	miR4512 down	NETosis	[246]

## Data Availability

The original contributions presented in this study are included in the article. Further inquiries can be directed to the corresponding authors.
